# Terrorism-Related Attacks in East Asia from 1970 through 2020

**DOI:** 10.1017/S1049023X23000109

**Published:** 2023-04

**Authors:** Heejun Shin, Attila J. Hertelendy, Alexander Hart, Derrick Tin, Fadi Issa, Ryan Hata, Gregory R. Ciottone

**Affiliations:** 1. BIDMC Disaster Medicine Fellowship, Department of Emergency Medicine, Beth Israel Deaconess Medical Center, Boston, Massachusetts USA; 2. Harvard Medical School, Boston, Massachusetts USA; 3. Department of Information Systems and Business Analytics, College of Business, Florida International University, Miami, Florida USA; 4. Department of Emergency Medicine, Hartford Hospital, Hartford, Connecticut, USA; 5. University of Connecticut School of Medicine, Farmington, Connecticut, USA; 6. Department of Critical Care, University of Melbourne, Melbourne, Australia

**Keywords:** Counter-Terrorism Medicine, Disaster Medicine, East Asia, epidemiology, terrorism

## Abstract

**Aim:**

This study aims to analyze and describe terrorism-related attacks in East Asia from 1970 through 2020.

**Background:**

East Asia consists of South Korea, North Korea, Singapore, Hong Kong, China, Japan, Taiwan, and Macao. According to the Global Terrorism Index (GTI) 2022, the impact of terrorism in East Asia is very low. However, the assassination of former Japanese Prime Minister Shinzo Abe on July 8, 2022 demonstrates that East Asia is not safe from terrorist attacks. This descriptive analysis of terrorist attacks in East Asia will help first responders, Emergency Medical Services (EMS), hospital-based medical providers, and policymakers establish a more refined hazard vulnerability assessment (HVA) framework and develop a Counter-Terrorism Medicine (CTM) mitigation, preparedness, response, and recovery plan.

**Methods:**

This is a descriptive observational study drawing data from the Global Terrorism Database (GTD) from January 1, 1970 through December 31, 2020. Epidemiology outcomes included primary weapon type, primary target type, the country where the incident occurred, and the number of total deaths and injured collected. Data from 2021 were not yet available at the time of this study. Results were exported into an Excel spreadsheet (Microsoft Corp.; Redmond, Washington USA) for analysis.

**Results:**

There were 779 terrorism-related events in East Asia from 1970 through 2020. In total, the attacks resulted in 1,123 deaths and 9,061 persons injured. The greatest number of attacks (371; 47.63%) occurred in Japan and the second most occurred in China (268; 34.4%). Explosives were the most used primary weapon type (308; 39.54%) in the region, followed by incendiary devices (260; 33.38%). Terrorist attacks drastically diminished from their peak of 92 in 1990, but there were additional peaks of 88 in 1996, 18 in 2000, 20 in 2008, and 36 attacks in 2014.

**Conclusions:**

A total of 779 terrorist attacks occurred from 1970 through 2020 in East Asia, resulting in 1,123 deaths and 9,061 injuries. Of those, 82.03% attacks occurred in Japan and China. Terrorist attacks drastically diminished since their peak in 1996, but there is an overall uptrend in attacks since 1999.

## Introduction

East Asia consists of the countries and regions of South Korea, Singapore, Hong Kong, China, Japan, Taiwan, Macao, and North Korea. According to the Global Terrorism Index (GTI) 2022, terrorism in East Asia is very low or no impact, as China (67^th^), Japan (71^th^), Taiwan (92^th^), South Korea (93^th^), and North Korea (93^th^) all rank in the lowest tiers of their rating system.^1^ However, through the assassination of former Japanese Prime Minister Shinzo Abe by a former Japan Maritime Self-Defence Force personnel on July 8, 2022, the world has witnessed that East Asia is not a terrorist-free region.^2^

There has been a significant amount of academic literature concerning individual, high profile attacks, such as the 1995 Tokyo Sarin attack in Japan.^3–6^ Previous literature concerning terrorism in the region has not included regional epidemiologic demographics.^7,8^ Specifically, there is no published study on the epidemiology of East Asia terrorist attacks covering 1970 through 2020.

Various studies have been published analyzing the open-source Global Terrorism Database (GTD) as the field of Counter-Terrorism Medicine (CTM) has developed.^9–14^ These studies deal with themes of mitigation, preparedness, and response in the fields of emergency and disaster medicine as it applies to terrorism from a global perspective.^9–13^ In addition, another systematic review study emphasized the importance of strategic communication, surveillance, planning, and training in preparation for mass-gathering terrorism, as demonstrated in the successful response to the Boston Marathon Bombing (Massachusetts USA) in 2013.^15^ Most of these studies have emphasized the analysis of region-specific counter-terrorism strategies contributing to hazard vulnerability analyses (HVAs), and this study was undertaken to advance that purpose.^9–13^

An analysis of past terrorism-related attacks in East Asia will help first responders, Emergency Medical Service (EMS) providers, hospital health providers, and administrative policymakers to establish HVAs and develop CTM management plans.

### Aim

This study aims to analyze and describe terrorism-related attacks in East Asia from 1970 through 2020.

## Methods

This is a descriptive study based upon a database source in design. Data collection was performed using a retrospective database search through the GTD.^14^ This database is open-access, with publicly available data collection methodology utilizing artificial intelligence that identifies events from news media around the world on a daily basis, and is confirmed by human evaluation of the events by the National Consortium for the Study of Terrorism and Responses to Terrorism (START; College Park, Maryland USA). The GTD defines terrorist attacks as: “The threatened or actual use of illegal force and violence by a non-state actor to attain a political, economic, religious, or social goal through fear, coercion, or intimidation.” The GTD database does not include acts of state-sponsored terrorism. The GTD contains no personal identifiers for victims and links specific events to open-source news articles.

The GTD was searched using the internal database search functions for all events which occurred in East Asia from January 1, 1970 - December 31, 2020. Years 2021 and 2022 were not yet available at the time of the study. Countries and regions classified under East Asia by the GTD for the study period include China, Hong Kong, Japan, Macau, North Korea, South Korea, and Taiwan.

Primary weapon type, primary target type, the country or region where the incident occurred, and the number of total deaths and injured were collated.

Results were exported into an Excel spreadsheet (Microsoft Corp.; Redmond, Washington USA) for analysis. Ambiguous events (this field is only systematically available with incidents occurring after 1997) were excluded when there was uncertainty as to whether the incident met any of the criteria for GTD inclusion as a terrorist incident. Attacks met inclusion criteria if they fulfilled the following three terrorism-related criteria, as set by the GTD.

These criteria are determined within the database and not by the authors:

- Criterion I: The act must be aimed at attaining a political, economic, religious, or social goal.
- Criterion II: There must be evidence of an intention to coerce, intimidate, or convey some other message to a larger audience (or audiences) than the immediate victims.
- Criterion III: The action must be outside the context of legitimate warfare activities (ie, the act must be outside the parameters permitted by international humanitarian law, particularly the admonition against deliberately targeting civilians or non-combatants).

The techniques used for extraction of study data were done by sorting East Asia region only, Criterion I, II, III met no Doubt Terrorism Proper (*doubtterr* coded as 0 or -9) from the raw database.

## Results

**Figure 1.**
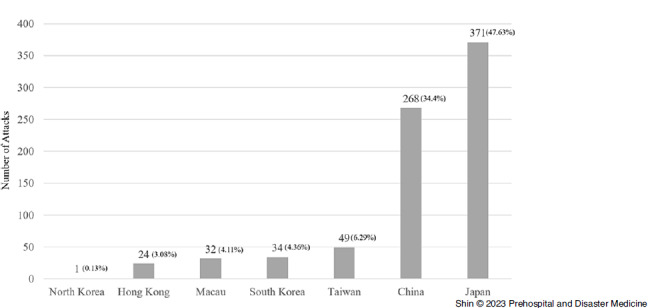
Attacks by Country or Region (Eastern Asia 1970-2020).

In total, 779 terrorist attacks were identified in the study period, resulting in 1,123 deaths and 9,061 wounded persons (Table 1). This equated to 1.44 deaths and 11.63 injuries per event. The greatest number of attacks occurred in Japan with 371 (47.63%), and the second most in China where 268 (34.4%) attacks occurred (Figure 1).

**Table 1.** Fatality and Injury Totals by Country

Variable	Total	Japan	China	Taiwan	South Korea	Macau	Hong Kong	North Korea
	**Terrorist Attack** **N = 779**	**Terrorist Attack** **N = 371**	**Terrorist Attack** **N = 268**	**Terrorist Attack** **N = 49**	**Terrorist Attack** **N = 34**	**Terrorist Attack** **N = 32**	**Terrorist Attack** **N = 24**	**Terrorist Attack** **N = 1**
**Death**								
Attacks Causing Death(s) (N)	740	342	262	49	31	31	24	1
Total Deaths (N)	1123	63	985	58	9	1	4	3
**Injury**								
Attacks Causing Injury (N)	734	344	255	48	31	31	24	1
Total Injuries (N)	9061	7002	1724	67	134	45	85	4

Explosives were the most common primary weapon type, as they were used in 308 (39.54%) attacks, followed by incendiary devices in 260 (33.38%) attacks (Table 2).

**Table 2.** Breakdown of Attacks by Primary Weapon Type

Primary Weapon Type	Number of Attacks	% of Total Attacks
Explosives	308	39.54%
Incendiary	260	33.38%
Melee	84	10.78%
Unknown	43	5.52%
Firearms	38	4.88%
Chemical	17	2.18%
Radiological	10	1.28%
Vehicle (excluding vehicle-borne explosives; ie, car or truck bombs)	8	1.03%
Fake Weapons	4	0.51%
Other	3	0.39%
Equipment Sabotage	3	0.39%
Biological	1	0.13%
Total	779	100.00%

Among the top five highest casualty totals, the top three occurred in Japan and the other two occurred in China (Table 3). These were also the two countries with the highest number of total attacks. The Aum Shinri Kyo Sarin attack in 1995 caused the most non-fatal injuries (5,500), with the China Urumqi attack in 2009 causing the most fatalities with 184 (Table 3).

**Table 3.** Top 5 Attacks based on Total Number of Casualties (Fatalities + Non-fatal Injuries)

Number	Date and Location	Perpetrator Group	Deaths	Injuries	Weapon	Target
1	1995-03-20 Japan, Tokyo	Aum Shinri Kyo	13	5500	Chemical attack by Sarin	Governmental subway system
2	1994-06-27 Japan, Tokyo	Aum Shinri Kyo	7	500	Chemical attack by Sarin gas	Private citizens and Judges
3	1995-04-19 Japan, Kanagawa	Unknown	0	671	Chemical attack by unknown gas	Railroad station
4	2009-07-05 China, Urumqi	Unknown	184	unknown	Armed assault (melee) by knives, wooden batons, bricks, and stones, and unknown incendiary device	Han Chinese civilians
5	2014-03-01 China, Yunnan	Uighur Separatists	33	143	Armed assault (melee) by knife or other sharp object	Kunming Railway Station

**Figure 2.**
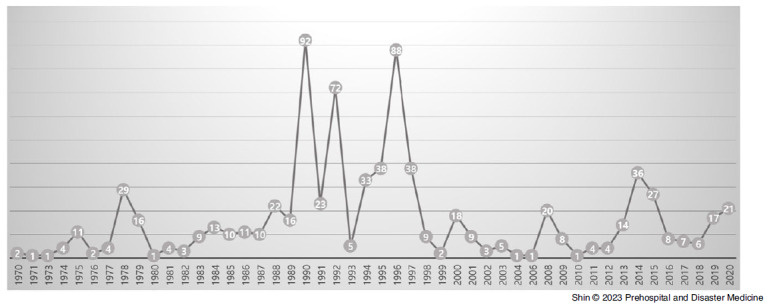
Number of Total Attacks in East Asia, by Year (1970-2020).

The yearly number of terrorist attacks hit a peak of 92 in 1990, and drastically decreased after 1996, but there were peaks of 18 in 2000, 20 in 2008, and 36 in 2014 in attacks since 1999. In 2019 and 2020, the last years for which data were available, there were again double-digit attacks (17 and 21, respectively); Figure 2.

## Discussion

There were 779 terrorism-related events in East Asia from 1970 through 2020. In total, the attacks resulted in 1,123 deaths and 9,061 non-fatal injuries. Explosives were the most used primary weapon type of all attacks in the region, followed by incendiary weapons. The number of terrorist attacks decreased sharply from 1996, but concerningly shows an increasing trend from 1999 to the present.

Even though the number of attacks by explosives is eighteen-times higher than by chemical weapons, study results showed that the frequency with which a type of weapon was used was not proportional to fatality and injury tolls. Chemical weapons attacks were responsible for the top three highest casualty tolls. No other single event created more than 200 total casualties. The highest casualty total from a single event came from the Aum Shinri Kyo attack using the nerve agent sarin. This attack targeted a government subway system in Tokyo (Japan).^3,4,16,17^ The terrorists took advantage of the subway system’s confined spaces with limited ventilation where a nerve agent was able to cause inhalation injuries maximizing the total number of casualties.^3,4,16–18^ As a result, it became one of the most significant terrorist attacks in world history, with 5,500 people injured.^3,4,16,17^ Nerve agents have also been used in individual assassination attempts such as in the 2018 Novichok poisoning.^19,20^ These findings are in line with previous research that the use of chemical weapons in terrorist attacks, although rare, remains a significant challenge and concern because of their ability to inflict massive numbers of casualties.^21^ Previous literature has determined that among global chemical terror attacks from 1970 through 2017, the most common routes of exposure were dermal-mucosal (43.8%), inhalational (37.2%), enteral (16.9%), parenteral (1.7%), and multiple (0.4%).^22^

The most frequent terrorist attack weapon type in East Asia was explosives. This follows the same trends seen in Western Europe, Eastern Europe, Middle East, and South Asia for similar study periods.^9,10,23,24^ On the contrary, incendiary weapons were the most frequent weapon type, and firearms were the second, in the United States from 2008 through 2018.^13^ This difference is due to the ease of accessibility of the population to assault weapons in the United States as opposed to other countries where firearms are more highly regulated.^25^

Despite the up-trending pattern of terrorist attacks in East Asia observed since 1999, there have been decreasing trends observed in the Middle East, Eastern Europe, South Asia, and Western Europe since 2016.^9,10,23,24^ This may in part be skewed by the different data collection methods inherent in the GTD, but still presents a point in need of further research.

Terrorism-related attack analysis of this kind benefits first responders, EMS providers, hospital-based providers, and policymakers and helps them to understand and prepare for their region-specific hazards, as well as to establish CTM management plans. Previous literature has emphasized the importance of strategic communication, monitoring, planning and preparation, training, and response for proper counter-terrorism management.^15^ As the results of this study show, the overwhelming number of casualties possible during chemical attacks demands that stakeholders create prevention and response plans for such events. This should include both chemical warfare agents, such as sarin, as well as hazardous industrial chemicals, such as those which create an irritant gas syndrome including chlorine or ammonia.^15,26–28^ Standard observational criteria or screening procedures should be in place to recognize sudden chemical attacks that may not be immediately apparent by observation of the toxidrome expressed by the victims.^15,26–28^

Rapid field decontamination protocols are also vital. Examples include Primary Response Incident Scene Management (PRISM) as detailed in United States federal guidelines, which includes steps designed to disrobe and dry decontaminate the patient, a ladder pipe system, and technical decontamination. An effective communication strategy should be implemented as well.^29^ The PRISM protocols attained a decontamination efficiency of almost 100% on exposed hair and skin sites on a previous large-scale study (Operation DOWNPOUR).^29^ It is also crucial to stockpile appropriate personal protective equipment and antidotes in the prehospital and hospital environments, as time to antidote after an exposure is a critical indicator of morbidity and mortality.^15,26–28^ Furthermore, there should be a focus on follow-up on-site management and expert support, such as CTM experts.^15^

## Limitations

The GTD is a comprehensive record of documented global terrorist events. It is maintained by the National Consortium for the Study of Terrorism and Responses to Terrorism (START) and is the basis for other terrorism-related measures, such as the GTI. Reliance wholly on the GTD is partially mitigated by confirmation with other lay sources and searches for other online sources, but if there are incidents not reported in the GTD, this could limit the accuracy of the findings. First, the GTD database represents a large convenience sample and therefore sampling error, selection bias, and lack of event reporting are limitations that should be reported. In addition, the website for the GTD database states that the accuracy of the data entered into the database is not confirmed by the database managers. Using pre-existing databases such as the GTD as a data source also inherently introduces potential challenges such as miscoding errors or data entry errors. Furthermore, the lack of a universally agreed-upon definition of the term terrorism can create inconsistencies between databases in the labeling of such events. Clear and detailed documentation of terrorist events is further hindered by restrictions on reporting, the lack of independent corroboration, and the lack of transparency within certain government sources. The infrastructure needed to report, detect, and investigate terrorism events is lacking in many parts of the world, leading to potential under-reporting of events.

## Conclusion

A total of 779 terrorist attacks occurred in East Asia from 1970 through 2020, resulting in 1,123 deaths and 9,061 injuries. Of those, 82.03% of attacks occurred in Japan and China. The frequency of terrorist attacks has overall diminished since peaking from 1990-1996, but there is an overall uptrend for the peaks in attacks since 1999.
